# Morbimortality assessment in abdominal surgery: are we predicting or overreacting?

**DOI:** 10.1186/s12893-021-01455-1

**Published:** 2022-01-18

**Authors:** Sebastian Valenzuela, Laura Niño, Danny Conde, Felipe Girón, Lina Rodríguez, David Venegas, Carlos Rey, Ricardo Nassar, Marco Vanegas, Daniel Jiménez

**Affiliations:** 1 Department of Surgery, Hospital Universitario Mederi, Calle 103a 21-93, 110111 Bogotá, D.C Colombia; 2 School of Medicine, Universidad del Rosario, Bogotá, Colombia; 3 School of Medicine, Universidad de los Andes, 111711 Bogotá, Colombia

**Keywords:** Risk prediction model, Mortality, Critical care, Laparotomy, Prognosis

## Abstract

**Background:**

High-risk surgical procedures represent a fundamental part of general surgery practice due to its significant rates of morbidity and mortality. Different predictive tools have been created in order to quantify perioperative morbidity and mortality risk. POSSUM (Physiological and Operative Severity Score for the enumeration of Mortality and morbidity) is one of the most widely validated predictive scores considering physiological and operative variables to precisely define morbimortality risk. Nevertheless, seeking greater accuracy in predictions P-POSSUM was proposed. We aimed to compare POSSUM and P-POSSUM for patients undergoing abdominal surgery.

**Methods:**

A retrospective observational study with a prospective database was conducted. Patients over 18 years old who complied with inclusion criteria between 2015 and 2016 were included. Variables included in the POSSUM and P-POSSUM Scores were analyzed. Descriptive statistics of all study parameters were provided. The analysis included socio-demographic data, laboratory values ​​, and imaging. Bivariate analysis was performed.

**Results:**

350 Patients were included in the analysis, 55.1% were female. The mean age was 55.9 ± 20.4 years old. POSSUM revealed a moderated index score in 61.7% of the patients, mean score of 12.85 points ± 5.61. 89.1% of patients had no neoplastic diagnosis associated. Overall morbidity and mortality rate was 14.2% and 7.1%. P-POSSUM could predict more precisely mortality (p < 0.00).

**Conclusions:**

The POSSUM score is likely to overestimate the risk of morbidity and mortality in patients with high/moderate risk, while the P-POSSUM score seems to be a more accurate predictor of mortality risk. Further studies are needed to confirm our results.

## Background

High-risk surgical procedures still carry considerable risk for patients in spite of advances in surgical technique and perioperative care thus making it of paramount importance the development of tools that predict postoperative outcomes for patient populations in specific surgical settings [1]. The optimal methods describe individual patient risk and account for a surgical procedure allowing preoperative optimization in individual postoperative settings [1]. This may lead to improve not only the decision-making process during the hospital stay, but also, allows clearer and more precise communication with patients and families in terms of expectations and possible outcomes [1]. Therefore, considering the growing need for better operative and medical decisions based on early identification of patients' high risk of morbimortality, multiple risk scores have emerged ranging from preoperative to postoperative scales [2].

Numerous studies have assessed its accuracy to predict life-threatening and non-life-threatening complications (defined by Clavien-Dindo classification 4–5) finding associations with significant statistical value. [2–4]. Copeland et al. developed the POSSUM (Physiological and Operative Severity Score for the enumeration of Mortality and morbidity) scoring system to help provide both retrospective and prospective analysis of the risk of postsurgical mortality and morbidity. The POSSUM score describes 18 factors in two component parts: 12 physiological factors (PS) and 6 operative factors (OS). From these values predicted mortality can be calculated [5] as it was developed as a tool to compare morbidity and mortality in a wide range of general surgical procedures to compare the performance of individual surgical units [1].

Given that the POSSUM scoring system was found to consistently overestimate the mortality rate in low-risk patients [6], in 1998 the need to adjust logistic regression analysis used in POSSUM scoring to better predict mortality resulted in a second scoring system using the same standard data, Portsmouth-POSSUM (P-POSSUM). This scoring system collects the same 18 physiological and operative parameters with a different calculation formula to predict mortality [7]. There on, both systems are used today helping guide utilization of health care resources for postoperative patients [8].

In terms of mortality, both Raut et al. and Mohammed et al. proved the accuracy of POSSUM and P-POSSUM scores in predicting mortality risk, with more than 80% of accuracy in selected patients [9, 10]. Additionally, Prabakaran et al. found that selected colorectal surgery patients have an increased prediction force, for that reason a subclass of POSSUM (CR-POSSUM) has been described in these populations [11]. Nevertheless, it has been described that score results shouldn’t be generalized to all patients that underwent surgical procedures and have to be personalized and analyzed according to each population [12].

Although good surgical technique is paramount in reducing adverse outcomes, these are dependent on the physiological state of patients, operative severity, and perioperative support devices [13]. The need for early identification of patients who are at high risk of mortality is present to outline and improve surgical outcomes and postoperative care [14]. The capability to predict life-threatening and high-cost complications may lead to an impact on the quality of life, and mortality rates in surgical patients. Hence, our aim is to describe the experience using POSSUM and P-POSSUM risk scores to predict morbidity and mortality in patients that underwent urgent abdominal surgical procedures in a high complexity hospital.

## Methods

Following Institutional Review Board approval, and after calculating a sample size of 350 using Dobson formula with a 95% confidence level. Ethical compliance with the Helsinki Declaration, current legislation on research Res. 008,430–1993 and Res. 2378–2008 (Colombia) and the International Committee of Medical Journal Editors (ICMJE) were ensured under our Ethics and Research Institutional Committee (IRB) approval.

### Patient’s description

The study includes all patients over 18 years old who underwent abdominal surgical procedures between 2015 and 2016 in a single institution. We exclude patients referred to our center with extra-institutional surgical management and those with missing data in the medical description. Morbidity was assessed by the clinical record, imagenologic findings, and surgical data, and classified according to the international Clavien-Dindo classification. Malignant disease was confirmed by histopathological analysis after surgery.

Data included patients' demographics, and all clinical, imaging, and blood chemistry needed for calculating POSSUM and P-POSSUM scores. Descriptive statistics of all study parameters were provided. Data were analyzed using IBM SPSS Statistics 25 software. Continuous data were summarized by their mean, standard deviation, median, minimum and maximum. Categorical data were summarized by their frequency and proportion. Bivariate analysis was performed. Qualitative variables were analyzed using chi-square statistics (Fisher's exact test when appropriate). Quantitative variables were analyzed, based on normality, with Spearman's or Pearson's association’s correlation coefficient accordingly. Bivariate analysis between qualitative and quantitative variables was performed using the Mann–Whitney test or the t-test for independent samples.

## Results

350 patients underwent urgent abdominal procedures, they were predominantly female (55.4%) with a mean age of 55.91 years old (Table 1). Among the clinical parameters measured, the mean systolic blood pressure was 125 mmHg and the average heart rate was 85.8 beats per minute, most of the patients did not have dyspnea (89.4%) or present signs or symptoms of heart failure (59.4%). However, an important group of patients (35.7%) received cardiovascular pharmacological management. In most cases, the Glasgow Score was not suggestive of head injury (14.9 ± 0.734) (Table 2).

**Table 1:** Demographic characteristics

Variable	Value
Sex	
Male	156 (44.5%)
Female	194 (55.4%)
Age (years) (mean ± SD)	55.91 ± 20.4

**Table 2:** Paraclinical and clinical characteristics

	Cardiac system n (%)	
	Without cardiac failure	208 (59.4%)
	Diuretic, digoxin, antianginous/antihypertensive	125 (35.7%)
	Peripheral edema, warfarin, borderline, cardiomegaly	15 (4.3%)
	Jugular engorgement, cardiomegaly	2 (0.6%)
POSSUM Physiological	Respiratory system (%)	313 (89.4%)
	Without dyspnea	17 (4.9%)
	Exercise dyspnea, mild COPD	17 (4.9%)
	Limiting dyspnea or moderate COPD	3 (0.9%)
	Dyspnea at rest or fibrosis/consolidation	
	Systolic blood pressure (mmHg) (mean ± SD)	125.8 ± 26.039
	Heart rate (bpm) (mean ± SD)	85.8 ± 16.819
	Glasgow coma score (mean ± SD)	14.9 ± 0.734
	Leukocytosis (× 10^9^/L) (mean ± SD)	11 818 ± 5324
	Hemoglobin (g/dl) (mean ± SD)	13.97 ± 14.15
	BUN (mg/dL) (mean ± SD)	17.54 ± 14.279
	Sodium (mEq/l) (mean ± SD)	138.3 ± 3.326
	Potassium (mEq/l) (mean ± SD)	3.96 ± 0.488
	EKG n (%)	
	Normal	341 (97.1%)
	Atrial fibrillation (HR 60–90 bpm)	5 (1.4%)
	Any other abnormal rhythm	4 (1.4%)
	Mean physiological score ± SD	19.27 ± 6.128
	Severity index n (%)	
	Moderate	216 (61.7%)
	Mayor	118 (33.7%)
	Mayor ( +)	16 (4.6%)
	Average number of procedures ± SD	1.54 ± 1.616
	Blood loss (cc) mean ± SD	75.63 ± 293.075
	Peritoneal fluid contamination n (%)	
Operative POSSUM	None	152 (43.4%)
	Minor (serous)	93 (26.6%)
	Local pus	54 (15.4%)
	Free fecaloid content, pus or blood	51 (14.6%)
	Presence of malignancy n (%) None	312 (89.1%)
	Primary	25 (7.1%)
	Nodal metastases	5 (1.4%)
	Distant metastasis	8 (2.3%)
	Surgical mode (%)	
	Elective	162 (46.3%)
	Urgent	56 (16.0%)
	Emergent (< 2 h)	132 (37.7%)
	Mean operative score ± SD	12.85 ± 5.61

Within blood chemistry, mean leukocyte count was 11,818 uL ± 5324, mean hemoglobin of 13.97 g/dl ± 4.15, normal renal function with a BUN of 17.54 mg/dl ± 14.279, electrolytes with sodium 138.3 mEq/l ± 3.326 and potassium 3.96 mEq ± 0.488, both within normal limits. Admission electrocardiogram was normal in 97.1% of patients. Mean physiological POSSUM was 19.27 ± 6.113points.

On the other hand, the mean operative POSSUM severity index was 12.85 ± 5.61 (moderate). 46.3% of patients underwent elective surgery, 16% urgent and 37.7% emergent. Mean blood loss was 75.63 cc. Peritoneal fluid had some change in appearance in half the patients, 26.6% was serous fluid, 15.4% had local pus, and 14.6% had free fecaloid content, pus, or blood. 89.1% did not present neoplasms, the remaining 10% had evidence of primary neoplasia (7.1%), nodal metastases (1.4%), or distant metastatic disease (2.3%). Mean operative POSSUM was 12.85 points ± 5.653.

Morbidity was analyzed using the Clavien-Dindo classification, 50 patients (14.2%) had some type of morbidity. Clavien-Dindo I was present in 2 patients that required hydro electrolytic replacement. In the case of Clavien-Dindo II, 10 patients required blood transfusions and the rest one’s parenteral nutrition. Clavien-Dindo IIIa was explained by patients that need percutaneous drainage of intra-abdominal collections, and type IIIb the remaining patients that need surgical intervention due to abdominal complications that conservative management fails. Clavien-Dindo IV was explained by patients who present respiratory failure and require orotracheal intubation. 7.1% mortality rate was documented (Clavien-Dindo V) (Table 3). Finally, we found a strong association between the P-POSSUM score and mortality (Pearson Coeff. 0.948 p = 0.000) (Table 4).

**Table 3:** Morbidity and mortality

Morbidity n (%)	
Clavien-Dindo I	2 (0.6%)
Clavien-Dindo II	16 (4.6%)
Clavien-Dindo IIIa	7 (2.0%)
Clavien-Dindo IIIb	23 (6.6%)
Clavien-Dindo IVa	1 (0.3%)
Clavien-Dindo IVb	1 (0.3%)
Mortality n (%)	25 (7.1%)

**Table 4:** Pearson's correlation

% Mortality	% Mortality	P-POSSUM
Pearson Correlation **p** **n**	1 350	0.948 0.000 349

## Discussion

Surgical risk assessment should be performed by a surgical team seeking a clear perspective not only for possible clinical outcomes but also in order to give patients and families a clear standpoint in terms of expectations [2]. Raw mortality and morbidity, though commonly reported, can be misleading due to differences in preoperative and intraoperative findings of patients [6, 15]. Although the good surgical technique is paramount in reducing adverse outcomes, the final result is a compound where the physiological state of the patient, operative severity, and peri-operative support services intervene [7]. Patient evolution and surgical complications depend on several factors: the quality of the surgical team, physiological status, and surgical stress among others [5]. Even though POSSUM and P-POSSUM scales have been widely used in Europe, their spread through America has not been equal [4].

The P-POSSUM scale was created in order to correct the elevated mortality risk obtained by POSSUM [3]; some studies showed an increased accuracy of P-POSSUM over POSSUM in mortality prediction, with near results with the observed mortality [9–12, 16]. Given that ​​P-POSSUM has to be correlated to the general condition of the local population for it to be effective [9–13], variability of results obtained is explained by lack of adjustments to population baseline characteristics that generate changes in predicted values. Among these characteristics are malnutrition and economic status; nevertheless literature, as our study, reports P-POSSUM scale has an increased accuracy to predict mortality compared with POSSUM in emergency surgery [12].

We assessed risk prediction in terms of morbidity and mortality by POSSUM and P-POSSUM scores. 61.7% of the patients underwent emergent surgical procedures with a moderate operative score, suggesting an increased risk of morbidity in the first 30 days after surgery. Nonetheless, actual morbidity in our population was significantly lower than predicted (14.2%), most of them classified as Clavien-Dindo II (4.6%) and IIIB (6.6%) were 46.6% of patients required a second surgical procedure, matching Gonzalez-Martinez et al. findings which sustained mortality and morbidity values calculated by POSSUM are overestimated [1, 17]. Stonelake et al. described 86 patients who underwent urgent laparotomy with a 77.9% of morbidity [2, 14], results higher than those found in our population (14.2%). Nonetheless, in terms of mortality, in our study, a higher mortality rate was documented compared to Stonelake et al. results (7.1% vs 5.81%) [2, 14]. This could be due to the not only surgery findings but also to the previous clinical status of the patients involved in our study, reflected in their higher physiological and surgical values.

Melendez et al., analyzed 513 patients for risk prediction using the POSSUM scale finding overestimated morbidity and mortality rates compared with actual values of 17.56% vs 10.33% and 4.5% vs 1.75% respectively [3, 18]. Additionally, P-POSSUM was more accurate, with a predicted mortality value of 1.6% vs an observed value of 1.75% [3, 18]. Mortality and morbidity outcomes in the Melendez cohort showed that the overestimation persisted to a lesser extent, which relates to our findings where values of mortality calculated by POSSUM score overestimated mortality rates and P-POSSUM score estimations had results closer to real (mortality rate 7.1% Vs P-POSSUM predicted mortality 6.31%), with a Pearson's coefficient 0.948 showing clear statistical correlation (p < 0.000). Additionally, most of the studies find discrepancies between expected and observed mortality with the use of POSSUM, but similar with P-POSSUM [1].

Despite the effectiveness of POSSUM and P-POSSUM scores, improvements are still needed because of the overinterpretation in morbidity and mortality, specifically in high complexity surgeries [8, 19–22]. Among the limitations of this study is its retrospective nature, data was obtained from previously collected clinical charts which may result in missing variables for some of the observations. Thus, more studies are needed to confirm our results.

## Conclusions

Overall, POSSUM seems to overestimate both morbidity and mortality. Whereas P-POSSUM appears to be closer to reality, it may be more accurate in estimating mortality in patients with moderate to high risk; adjustment to baseline characteristics of the population could increase the accuracy. Even though P-POSSUM seems like a feasible score to predict morbimortality in abdominal surgery, further studies are needed to confirm our results.

## Data Availability

Dataset and materials used during the current study are available from the corresponding author on request.

## Abbreviations

POSSUM: Physiological and Operative Severity Score for the enumeration of Mortality and morbidity
P-POSSUM: Portsmouth-Physiological and Operative Severity Score for the enumeration of Mortality and morbidity
PS: Physiological factors
OS: Operative factors
COPD: Chronic obstructive pulmonary disease
